# Enhanced Immune Responses with Serum Proteomic Analysis of Hu Sheep to Foot-and-Mouth Disease Vaccine Emulsified in a Vegetable Oil Adjuvant

**DOI:** 10.3390/vaccines8020180

**Published:** 2020-04-15

**Authors:** Xuemei Cui, Yong Wang, Ran Guan, Meiqian Lu, Lijia Yuan, Wei Xu, Songhua Hu

**Affiliations:** Department of Veterinary Medicine, College of Animal Sciences, Zhejiang University, 866 Yu Hang Tang Rd, Hangzhou 310058, China; maycui@zju.edu.cn (X.C.); wyuan526@zju.edu.cn (Y.W.); 11517009@zju.edu.cn (R.G.); 21717084@zju.edu.cn (M.L.); 11617034@zju.edu.cn (L.Y.); zhihong0902@zju.edu.cn (W.X.)

**Keywords:** adjuvant, soybean oil, vitamin E, ginseng saponins, foot-and-mouth disease, proteomic

## Abstract

Our previous study demonstrated that a vegetable oil consisting of soybean oil, vitamin E, and ginseng saponins (SO-VE-GS) had an adjuvant effect on a foot-and-mouth disease (FMD) vaccine in a mouse model. The present study was to compare the adjuvant effects of SO-VE-GS and the conventional ISA 206 on an FMD vaccine in Hu sheep. Animals were intramuscularly (i.m.) immunized twice at a 3-week interval with 1 mL of an FMD vaccine adjuvanted with SO-VE-GS (n = 10) or ISA 206 (n = 9). Animals without immunization served as control (n = 10). Blood was sampled prior to vaccination and at 2, 4, 6, and 8 weeks post the booster immunization to detect FMD virus (FMDV)-specific IgG. Blood collected at 8 weeks after the booster was used for the analyses of IgG1 and IgG2, serum neutralizing (SN) antibody, IL-4 and IFN-γ production, and proteomic profiles. The results showed that IgG titers rose above the protection level (1:128) in SO-VE-GS and ISA 206 groups after 2 and 4 weeks post the booster immunization. At 6 weeks post the booster, the ISA 206 group had 1 animal with IgG titer less than 1:128 while all the animals in the SO-VE-GS group retained IgG titers of more than 1:128. At 8 weeks post the booster, 6 of 9 animals had IgG titers less than 1:128 with a protective rate of 33.3% in the ISA 206 group, while only 1 of 10 animals had IgG titer less than 1:128 with a protective rate of 90% in the SO-VE-GS group, with statistical significance. In addition, IgG1, IgG2, SN antibodies, IL-4, and IFN-γ in the SO-VE-GS group were significantly higher than those of the ISA 206 group. Different adjuvant effects of SO-VE-GS and ISA 206 may be explained by the different proteomic profiles in the two groups. There were 39 and 47 differentially expressed proteins (DEPs) identified in SO-VE-GS compared to the control or ISA 206 groups, respectively. In SO-VE-GS vs. control, 3 immune related gene ontology (GO) terms and 8 Kyoto Encyclopedia of Genes and Genomes (KEGG) pathways were detected, while 2 immune related GO terms and 5 KEGG pathways were found in ISA 206 vs. control. GO and KEGG analyses indicated that ‘positive regulation of cytokine secretion’, ‘Th1/Th2 cell differentiation’, and ‘Toll-like receptor signaling pathways’, were obviously enriched in the SO-VE-GS group compared to the other groups. Coupled with protein–protein interaction (PPI) analysis, we found that B7TJ15 (MAPK14) was a key DEP for SO-VE-GS to activate the immune responses in Hu sheep. Therefore, SO-VE-GS might be a promising adjuvant for an FMD vaccine in Hu sheep.

## 1. Introduction

Foot-and-mouth disease (FMD) is a highly contagious viral disease that spreads rapidly among cloven hoofed animals, causing large economic losses after outbreaks [1,2,3,4]. The disease is caused by the FMD virus (FMDV), which includes seven serotypes (A, C, O, South African Territories (SAT) 1, SAT 2, SAT 3, and Asia 1) and multiple subtypes within each serotype [5]. Among the seven serotypes of FMDV, type O is the most prevalent worldwide [6,7]. FMD is listed by the International Organization of Animal Health (OIE) [8] as one of the world’s most important animal diseases [9,10]. 

The susceptible animals include zebu, cattle, yak, swine, camels, sheep, goats, and deer [11,12]. Among them, sheep are an economically important susceptible species [13]. FMD causes fever and blister-like lesions or vesicles on the oral cavity, nostrils, muzzle, coronary bands, teats, udder, and interdigital spaces [14,15]. As a result, the affected animals show excessive salivation and lameness. In sheep and goats, the signs are usually less visible or mild and difficult to diagnose [16,17]. The morbidity rate approaches 100% in susceptible animals. However, the disease is seldom fatal [18]. Although sheep and goats may not clinically manifest typical FMD signs, they can excrete a large amount of FMDV with much longer duration than do cattle [19,20]. Therefore, the small ruminant may represent the main FMD-susceptible livestock and play a significant role in spreading the infection during outbreaks in some areas of the world [21,22]. Usually, a comprehensive approach including surveillance, limitation of movement in susceptible animals, slaughter of diseased animals, and vaccination are recommended in FMD endemic areas [23,24]. Horsington et al. observed that immunization of sheep with an FMD vaccine induced a neutralizing antibody response and protected the animals from infection after challenge [17].

Hu sheep is a local breed that originated in the Tai Lake Valley in Southeast China [25,26]. It has become an important economical animals in most areas of China as it has advantages of early sexual maturation, good reproductive performance with multiple offspring, genetic stability, and good adaptability to the environment [27]. To control FMDV infection, immunization of Hu sheep with type O FMD vaccine is compulsory in China [10,28]. Although the efficacy of immunization with FMD vaccines in sheep and other sheep-like species have been reported [29,30], no reports were found regarding the efficacy of immunization with FMD vaccine in Hu sheep. To improve the immune response, commercially available FMD vaccines are usually adjuvanted with Montanide ISA 206, a mineral oil-based adjuvant [31]. Our previous study demonstrated that a vegetable oil containing both vitamin E and ginseng saponins (SO-VE-GS) had adjuvant effect and significantly improved the immune response induced with an FMD vaccine in mice [32]. VE included in vaccines to improve the immunostimulation has been reported in other studies [33,34]. Morel et al. found that VE was required for an adjuvant AS03 to achieve the enhanced antibody response [33]. Karlsson et al. reported that an adjuvant Diluvac Forte^®^ containing VE enhanced the immunogenicity of a naked DNA vaccine encoding influenza proteins [35]. The present study was designed to evaluate the adjuvant effect of ISA 206 and SO-VE-GS on the immune response induced by FMD vaccines in Hu sheep by measuring the antibody responses, determining serum IFN-γ and IL-4, as well as analyzing proteomics.

## 2. Materials and Methods

### 2.1. Animals

From Huzhou Miemie Sheep Farm (Huzhou, China), a commercial farm, 29 animals that had received no immunization of vaccines were selected for this study and they were 55 ± 5 days old with the bodyweight of 16.50 ± 0.15 kg. The animals were housed in cages, and feed and water were supplied ad libitum throughout the experiment. All trials pertaining to animal use and their care strictly followed the Guidelines of Laboratory Animals of Zhejiang University and all the protocols were approved by Zhejiang University Animals Ethics Committee (ZJU20160377) on 4 March 2016.

### 2.2. Vaccine Preparation

SO-VE-GS was prepared from soybean oil (SO) (Zhejiang Tian Yu Shan Medicinal Co. Ltd., Zhejiang, China), vitamin E (VE) (Sigma-Aldrich no. T3251, Saint Louis, MO, USA), and standardized ginseng saponins (GS) (Hongjiu Ginseng Industry Co. Ltd., Jilin, China). Montanide ISA 206 adjuvant was the product of Seppic Co. Ltd. (Shanghai, China). Inactivated FMDV (type O, strain OHM/02) was kindly supplied by Tian Kang Biotech Co. Ltd. (Xinjiang, China). The virus was inactivated with binary ethylenimine, and 146S whole inactivated particles were used for vaccine preparation. The antigen was diluted in physiological saline solution to a required concentration and then added to ISA 206 in a ratio of 50:50 (w/w) or SO-VE-GS in a ratio of 50:50 (v/v). To prepare the SO-VE-GS adjuvanted vaccine [32], the mixture was emulsified at a shear rate of 14,500 rpm for 10 min at 30 °C (IKA T10 basic, ULTRA-TURRAX1, Staufen, Germany) to form an emulsion of oil-in-water (O/W). ISA 206 adjuvanted vaccine was prepared according to the manufacturer’s instructions. Briefly, ISA 206 and antigen aqueous phase were warmed to 31 ± 1 °C separately and then mixed under 350 rpm agitation for 5 min to form an emulsion of water-in-oil-in-water (W/O/W). The two prepared FMD vaccines contained 4 μg of 146S virus particles per mL. The 146S antigen represents purified inactivated FMDV particles (sedimentation coefficient of 146). 

### 2.3. Immunization and Sampling

**Figure 1:**
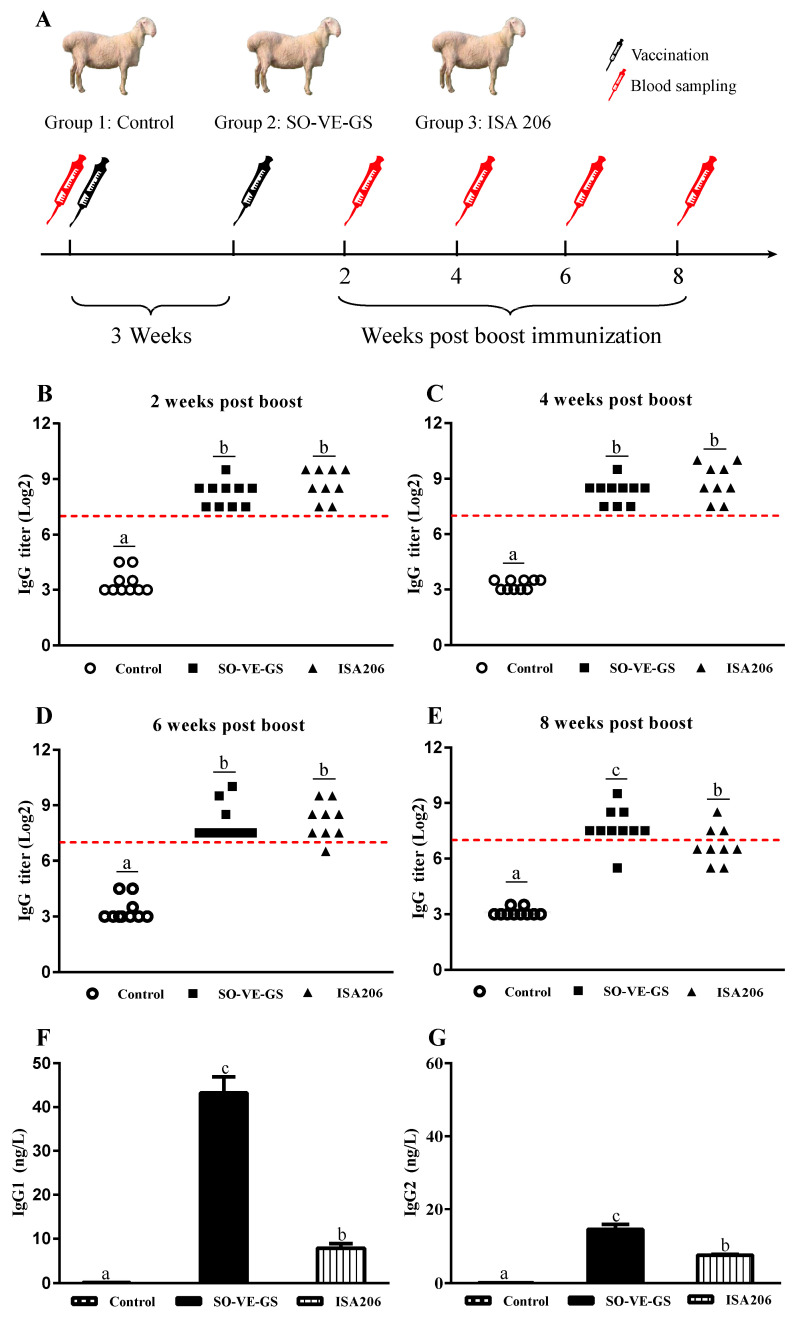
Serum Foot-and-mouth disease (FMD) virus (FMDV)-specific antibody response. (**A**) Experimental design: Hu sheep were intramuscularly (i.m.) immunized twice at a 3-week interval with FMD vaccine emulsified in a vegetable oil consisting of soybean oil, vitamin E, and ginseng saponins (SO-VE-GS) (n = 10) or ISA 206 (n = 9), and sheep without immunization served as control (n = 10). Blood samples were taken prior to vaccination and at 2, 4, 6, and 8 weeks after the booster immunization to detect serum FMDV-specific IgG. (**B**–**E**) FMDV-specific IgG titers determined at 2, 4, 6, and 8 weeks post the booster; dotted horizontal line was at an IgG titer of 1:128, indicating the minimum protection titer. (**F**–**G**) IgG1 and IgG2 measured at 8 weeks post the booster. The values are presented as mean ± SE. Data with different letters are statistically different (*P* < 0.05).

Animals were divided into three groups. Group 1 was not treated and was used as a control (*n* = 10), groups 2 (*n* = 10) and 3 (*n* = 9) were intramuscularly (i.m.) immunized twice at a 3-week interval with 1 mL of FMD vaccine adjuvanted with SO-VE-GS or ISA 206, resepctively. Blood samples were taken prior to vaccination and at 2, 4, 6, and 8 weeks after the booster immunization to detect serum FMDV-specific IgG. Blood collected at 8 weeks after booster immunization was also used to analyze IgG isotypes, serum neutralizing (SN) antibody, cytokine production, and proteomic analysis (Figure 1A). 

### 2.4. Analysis of FMDV-Specific Antibody and Isotypes

Serum FMDV-specific antibody titers were determined by a liquid phase blocking (LPB) ELISA kit (Lanzhou Veterinary Research Institute, Lanzhou, China) according to the manufacturer’s instructions [2,36]: LPB-ELISA antibody titers ≥ 7 log2 (1:128) were considered to have protection (Figure S1). Briefly, 50 µL of two-fold serial dilutions of serum samples and 50 µL of FMDV antigen (1:20 dilution) were added to a U-bottomed 96-well plate and incubated for 1.5 h at 37 °C. The mixtures then were transferred into a 96-well ELISA plate precoated with rabbit anti-FMDV polyclonal antibody and incubated for 1 h at 37 °C. After five washes, plates were incubated with 50 µL of guinea pig antiserum against FMDV O serotype for 30 min at 37 °C. Then, 50 µL of rabbit anti-guinea pig IgG/HRP was added to the 96-well plate after a washing step (total of five washes with PBST) and incubated for 30 min at 37 °C. The plate was washed and 50 µL of the substrate/chromophore mixture was added to each well, and the plate was incubated for 15 min at 37 °C in the dark. Finally, 50 µL of stop solution was added to each well, and absorbance at 492 nm was measured within 15 min of stopping the reaction. Serum FMDV-specific IgG1 and IgG2 isotype antibodies were measured by an indirect ELISA kit (Kanglang Biotechnology Co., Ltd, Shanghai, China). 

### 2.5. Serum Neutralizing Antibody Test 

Serum neutralizing (SN) antibody was assayed according to the method described in the OIE manual [37]. Heat inactivated serum samples were diluted two-fold from 1:16 to 1:2048 in 96-well microtiter plates (50 µL/well), then 50 µL of FMDV suspension that contained 100 TCID_50_ was added to each well. The plate was gently agitated to mix the contents and then incubated at 37 °C in 5% CO_2_ for 1 h. After that, 100 µL of baby hamster kidney (BHK21) cells (5.0 × 10^5^ cells/mL) were added into each well, and incubated for another 72 h. The wells were examined for cytopathic effects, and SN antibody titer was calculated as the reciprocal of the highest serum dilution to neutralize 100 TCID_50_ of the virus. The Reed and Muench formula was adopted to calculate of SN titer [38]. Results were expressed as the Log_10_ SN titer [2]. Sera with titers > 1.5 Log_10_ are considered positive (OIE Manual of Diagnostic Tests and Vaccines for Terrestrial Animals 2019) [17].

### 2.6. Determination of Serum Cytokine Levels

Serum gamma interferon (IFN-γ) and interleukin 4 (IL-4) levels were measured using ELISA kits (RayBiotech, Inc, GA, USA). Cytokine levels were evaluated by interpolation of cytokine standard curves, according to the manufacturer’s instructions.

### 2.7. Proteomic Analysis

To explore the molecular mechanisms involved in the two adjuvants, tandem mass tags (TMT) technology was used for quantitative proteomic analysis according to previously described method [39,40]. Serum (0.5 mL) was prepared from each animal and pooled in the same group, which was then divided into three samples used for the test. Basically, protein was extracted from the serum samples of the sheep (n = 3/group), and then reconstituted in dissolution buffer, denatured, reduced, alkylated, and trypsinized. After centrifuging, the supernatant was collected for determination of protein concentration by bicinchoninic acid (BCA) method. After that, protein was digested following a standard procedure and the resulting peptides were labeled using the 10-plex TMT reagent according to the manufacturer’s instructions (Thermo fisher, Art.No.90111, MA, USA). 

Each sample was injected for nano LC-MS/MS analysis in a Q Exactive mass spectrometer that was coupled with Easy-nLC 1200 (Thermo Fisher Scientific, USA) [41]. The peptide mixture (2 μg) was loaded onto the C18 column (75 μm × 25 cm, MA, USA) in buffer A (2% acetonitrile and 0.1% formic acid) and separated with a linear gradient of buffer B (80% acetonitrile and 0.1% formic acid) at a flow rate of 300 nL/min. The electrospray voltage of 1.8 kV versus the inlet of the mass spectrometer was used. Q Exactive mass spectrometer was operated in the data-dependent mode to switch automatically between MS and MS/MS acquisition. Full MS scans ranging from 350 to 1500 m/z were acquired at a resolution of 60,000 (at 200 m/z) with an automatic gain control (AGC) target value of 3 × 10^6^ and a maximum ion injection time of 20 ms. The 40 most abundant precursor ions from the full MS scan were selected for fragmentation using higher energy collisional dissociation (HCD) at a resolution of 15,000 (at 200 m/z) with an AGC target value of 1 × 10^5^, a maximum ion injection time of 45 ms, a normalized collision energy of 32%, an intensity threshold of 8.3 × 10^3^, and the dynamic exclusion parameter of 60 s. 

### 2.8. Differential Expression Proteins (DEPs) Analysis

Using the Student’s t-test function in R to calculate the *P* value of significant difference between sample groups. In this project, differential expression proteins (DEPs) screening criteria were *P* < 0.05 and (FC > 1.20 or FC < 0.83). Goatools (https://github.com/tanghaibao/GOatools) software was exploited to identify statistically significantly enriched gene ontology (GO) terms using Fisher’s exact test. The purpose of performing FDR (False Discovery Rate) correction is to reduce the Type-1 errors by the Bonferroni method (multiple hypothesis test method). After multiple testing correction, GO terms with corrected and *P* values ≤ 0.05 were considered significantly enriched in DEPs. The Kyoto Encyclopedia of Genes and Genomes (KEGG) pathway significant enrichment analysis enhances the reliability of the study and identifies the biological processes that are most relevant to biological phenomena in which the DEPs are involved. KOBAS 2.0 (http://kobas.cbi.pku.edu.cn/home.do) is exploited to identify statistically significantly enriched pathways using Fisher’s exact test. The purpose of performing FDR correction is to reduce the Type-1 error by BH (Benjamini and Hochberg, multiple hypothesis test method). KEGG pathways with corrected *P* values ≤ 0.05 were considered significantly enriched in DEPs. After multiple testing correction, we choose pathways with *P* values ≤ 0.05 as significantly enriched in DEGs. Sensitivity analysis was performed to evaluate the robustness of KEGG and GO. Briefly, one protein was deleted from a specific item, and the KEGG and GO tests were re-run. Then, this protein was added back and a different protein was deleted from the list, and the tests re-run. We compared the results before and after deleting to see whether some items always popped up or not. If the detection of an item was reliant on a small number of proteins, then this result was reliable.

### 2.9. Statistical Analysis

SPSS 20.0 software (SPSS Inc., Chicago, IL, US) was adopted for data analysis. The data were analyzed by one-way ANOVA with the least significant difference test. Results were expressed as mean ± SE. *P* < 0.05 was considered statistically significant.

## 3. Results

### 3.1. FMDV-Specific Antibody Response

Serum FMDV-specific IgG responses were detected by liquid phase blocking ELISA (LPB-ELISA). According to the Lanzhou Veterinary Research Institute, Chinese Academy of Agricultural Sciences, serum with LPB-ELISA IgG titers ≥ 1:128 is considered to have protection against FMDV infection. Before immunization, serum IgG titers were < 1:16 in all three groups. Two and 4 weeks after the booster immunization, all animals in both SO-VE-GS and ISA 206 groups had IgG titers above the level for protection (Figure 1B-C). Six weeks after the booster immunization, the ISA 206 group had one animal with serum IgG titer less than 1:128 while all the animals in the SO-VE-GS group had serum IgG titers more than 1:128 (Figure 1D). Eight weeks after the booster immunization, 6 of 9 animals had IgG titers less than 1:128 with a protective rate of 33.3% in the ISA 206 group while only 1 of 10 animals had an IgG titer less than 1:128 with a protective rate of 90% in the SO-VE-GS group (Figure 1E), and IgG titer in the SO-VE-GS group was significantly higher than that of the ISA 206 group (Figure S2). Analysis of IgG isotypes showed that IgG1 and IgG2 in the SO-VE-GS group were significantly higher than those of the ISA 206 group (Figure 1F–G).

### 3.2. Serum Neutralizing Antibody Assay

A titer of 1:45 or more of the final serum dilution in the serum/virus mixture is regarded as positive. A titer of less than 1:16 is considered to be negative (OIE Manual of Diagnostic Tests and Vaccines for Terrestrial Animals 2019). Serum neutralizing (SN) antibody titer was analyzed 8 weeks after the booster immunization. A significantly higher SN antibody titer was detected in the SO-VE-GS group than in the other two groups, having titers from 1:49 to 1:328, while no detectable SN titers were detected in either the ISA 206 or control groups (< 1:16) (Table 1) (Figure S3).

### 3.3. Cytokines Assay

**Figure 2:**
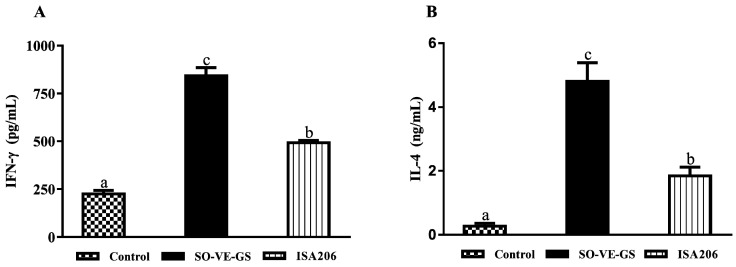
Serum IFN-γ and IL-4. Hu sheep were i.m. immunized twice at a 3-week interval with FMD vaccine emulsified with SO-VE-GS (n = 10) or ISA 206 (n = 9), and sheep without immunization served as a control (n = 10). Blood samples were collected at 8 weeks post the booster for analysis of IFN-γ (**A**) and IL-4 (**B**). The values are presented as mean ± SE. Bars with different letters are statistically different (*P* < 0.05).

To investigate the cellular immune response in the three groups, blood samples were collected from all the sheep at 8 weeks post the booster to test serum cytokine levels. Figure 2 shows that sheep immunized with FMD vaccine had significantly higher IFN-γ and IL-4 than the control group. However, the highest levels of IFN-γ (Figure 2A) and IL-4 (Figure 2B) were detected in the sheep immunized with FMD vaccine adjuvanted with SO-VE-GS. The higher cytokine levels might be due to the chronic inflammation induced by SO-VE-GS.

### 3.4. Protein Identification and Quantitation

**Figure 3:**
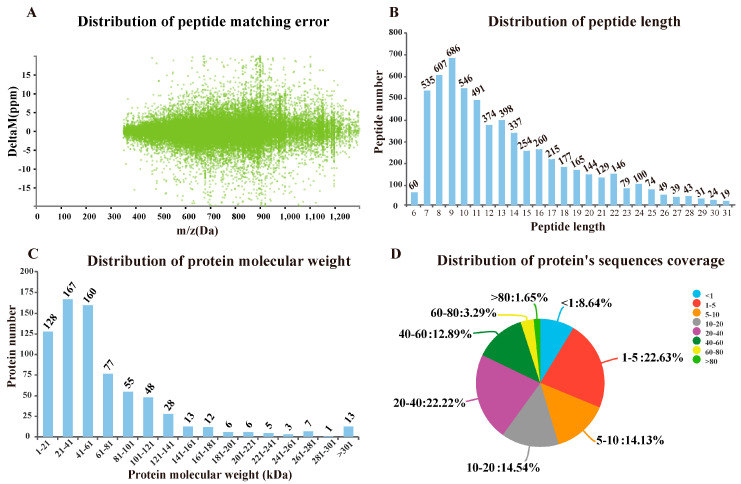
Identification and quantitation of the proteome. (**A**) The distribution of peptide matching error. (**B**) The distribution of peptide length. (**C**) The distribution of protein molecular weight. (**D**) The distribution of protein’s sequences coverage. The number outside the fan indicates the coverage of the protein number in this interval.

Using Q Exactive mass spectrometer analysis, a total of 1330 proteins were found to match 5020 unique spectrum hits in the Proteome Discoverer (Figure S4). As shown in Figure 3A, the peptide matching error was between ± 20 ppm and the peptide length was around in 7–20 aa, among which the 7–11 aa interval was the peak area (Figure 3B). The molecular weights of the most-identified proteins are exhibited in Figure 3C, and 86% of the protein sequence coverage was at 1–60% (Figure 3D). 

### 3.5. Differential Expression Proteins (DEPs) Analysis

**Figure 4:**
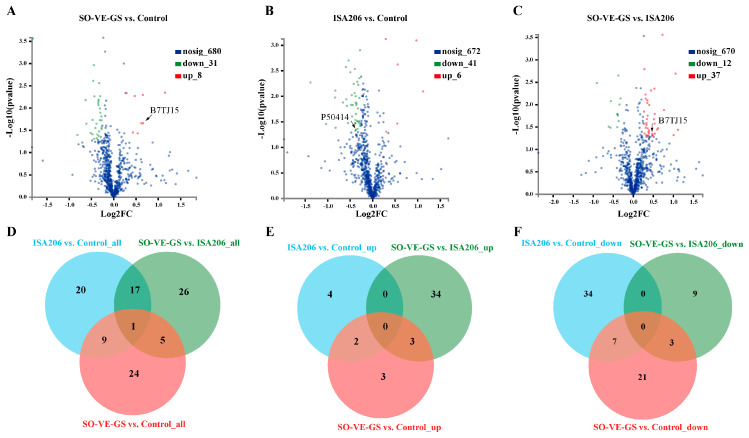
Differentially expressed proteins (DEPs) in three comparisons. Blood samples (n = 3) were collected at 8 weeks after immunization of Hu sheep with FMD vaccines adjuvanted with SO-VE-GS or ISA 206, and animals without immunization served as a control; sera were used for proteomic analysis. (**A**–**C**) Volcano plots of DEPs in three comparisons: SO-VE-GS vs. control, ISA 206 vs. control, and SO-VE-GS vs. ISA 206. MAPK14 (B7TJ15) and TGF-β1 (P50414) were labeled. (**D**–**F**) Venn diagrams of differentially expressed proteins enriched by gene ontology (GO) analysis (*P* < 0.05, fold change > 1.2 or < 0.83 = Log_2_FC > 0.26 or Log_2_FC < −0.26).

The tandem mass tags (TMT)-labelling-based quantitative proteomic approach was used to identify differentially expressed protein (DEP) profiles in serum. Compared with the control group, 39 DEPs were identified in the SO-VE-GS group with 8 up- and 31 downregulated proteins, and in the ISA 206 group, 47 DEPs were identified with 6 up- and 41 downregulated proteins (Figure 4A,B). Compared to the ISA 206 group, 49 DEPs were identified in the SO-VE-GS group with 37 up- and 12 downregulated proteins (Figure 4C). To identify common and specific DEPs in each of the group pairs, Venn diagrams were generated. Figure 4D shows that 20, 26, and 24 DEPs were found specifically in each of the group pairs, and only 1 DEP, W5PGT9, an uncharacterized protein, was shared in all three groups. In addition, 17, 5, and 9 DEPs were commonly shared in each of the two group pairs. Among them, up- and downregulated DEPs were also analyzed in Venn diagrams (Figure 4E,F); there was no commonly shared up- or downregulated protein in all three groups. When compared with the control group, 2 up- (W5Q5J4, IL1RAP) and 7 downregulated (CAPNS, DAG1, CADM1, MGC137014, PAM, CES, Bt.48679) proteins were shared by the two adjuvant groups. (Protein relating common names to UniProt identifiers are shown in Table S1).

### 3.6. Gene Ontology (GO) Enrichment Analysis of DEPs

**Figure 5:**
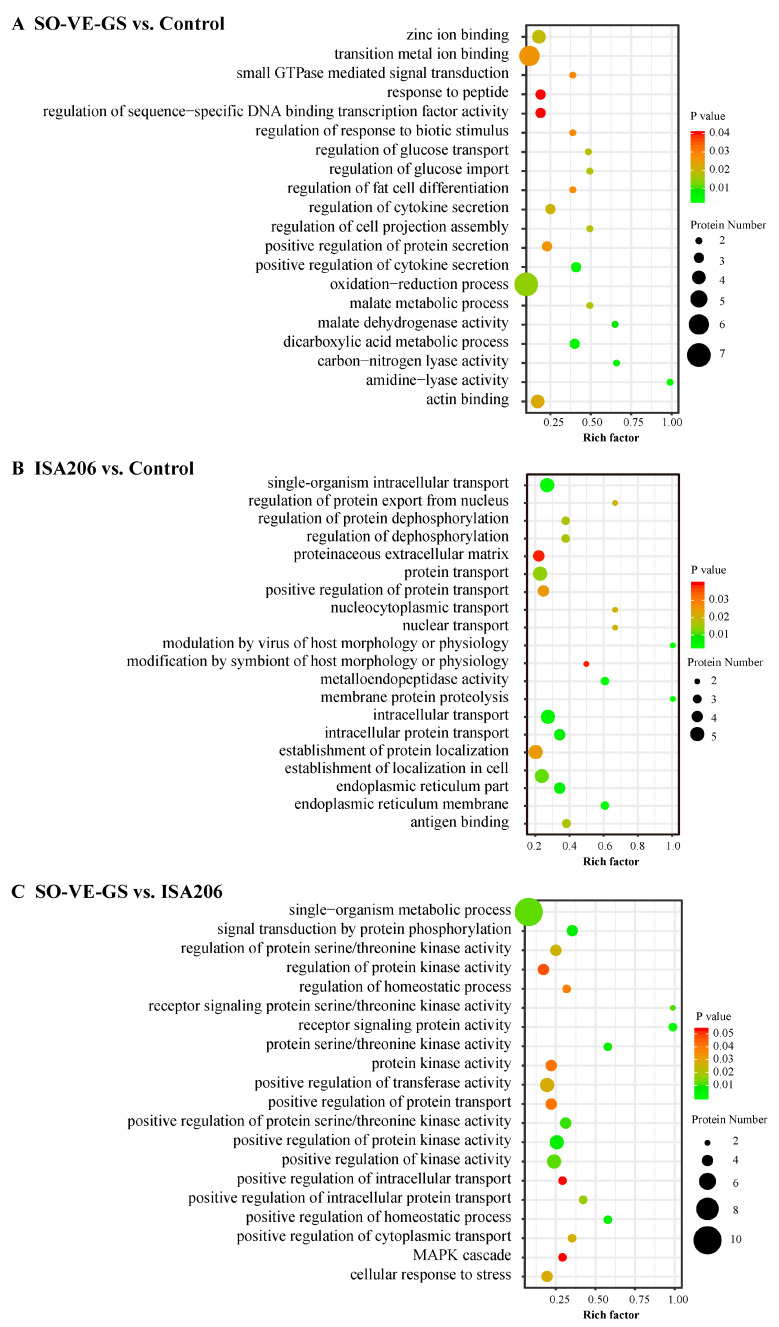
GO functional enrichment analysis. DEPs of (**A**) SO-VE-GS vs. control, (**B**) ISA 206 vs. control, and (**C**) SO-VE-GS vs. ISA 206 were used for GO functional enrichment analysis. The x-axis represents the value of the rich factor. The y-axis represents the enrichment of GO terms. The size of the dot represents the number of DEPs. The color represents corrected *P* value.

The functional enrichment of DEPs was based on GO (gene ontology) enrichment analysis [42], which includes three categories: biological process (BP), molecular function (MF), and cellular component (CC). The top 20 enriched GO terms for DEPs are reported. Sensitivity analysis indicated that these GO terms were reliable (Table S2). In the comparison of SO-VE-GS vs. control, 14 GO terms were assigned to the BP ontology and 6 were assigned to the MF ontology. As shown in Figure 5A, the immune-related GO terms such as positive regulation of cytokine secretion including IL1RAP, CADM1, and MAPK14, oxidation-reduction processes including PAM, MDH1, C5IJ88, MAPK14, C0LQH2, ME3, and W5NUW3, and regulation of responses to biotic stimuli including HRG and MDH1 were significantly identified in SO-VE-GS. In the ISA 206 vs. control comparison, among the 20 identified GO terms 3, 2, and 15 were of the CC, MF, and BP ontology, respectively. As Figure 5B shows, significantly higher expressed proteins related to the endoplasmic reticulum membrane including ERP44, IGFBP4, and HSP90B1, antigen binding including YWHAE, TGF-β1, and W5PGE9, and modulation by virus of host morphology or physiology including TGF-β1 and DAG1 GO terms were notably enriched in the ISA 206 group. In the comparison pf SO-VE-GS vs. ISA 206, 16 and 4 GO terms were assigned to the BP and MF ontology, respectively. The most significantly enriched GO terms were receptor signaling protein activity including ERBB3, MAP2K5, and MAPK14, GO terms of positive regulation of homeostatic process including FCGR3A, MAPK14, and CEMIP, and receptor signaling protein serine/threonine kinase activity including MAP2K5 and MAPK14 were also significantly activated (Figure 5C).

### 3.7. Kyoto Encyclopedia of Genes and Genomes (KEGG) Pathway Enrichment Analysis of DEPs

**Figure 6:**
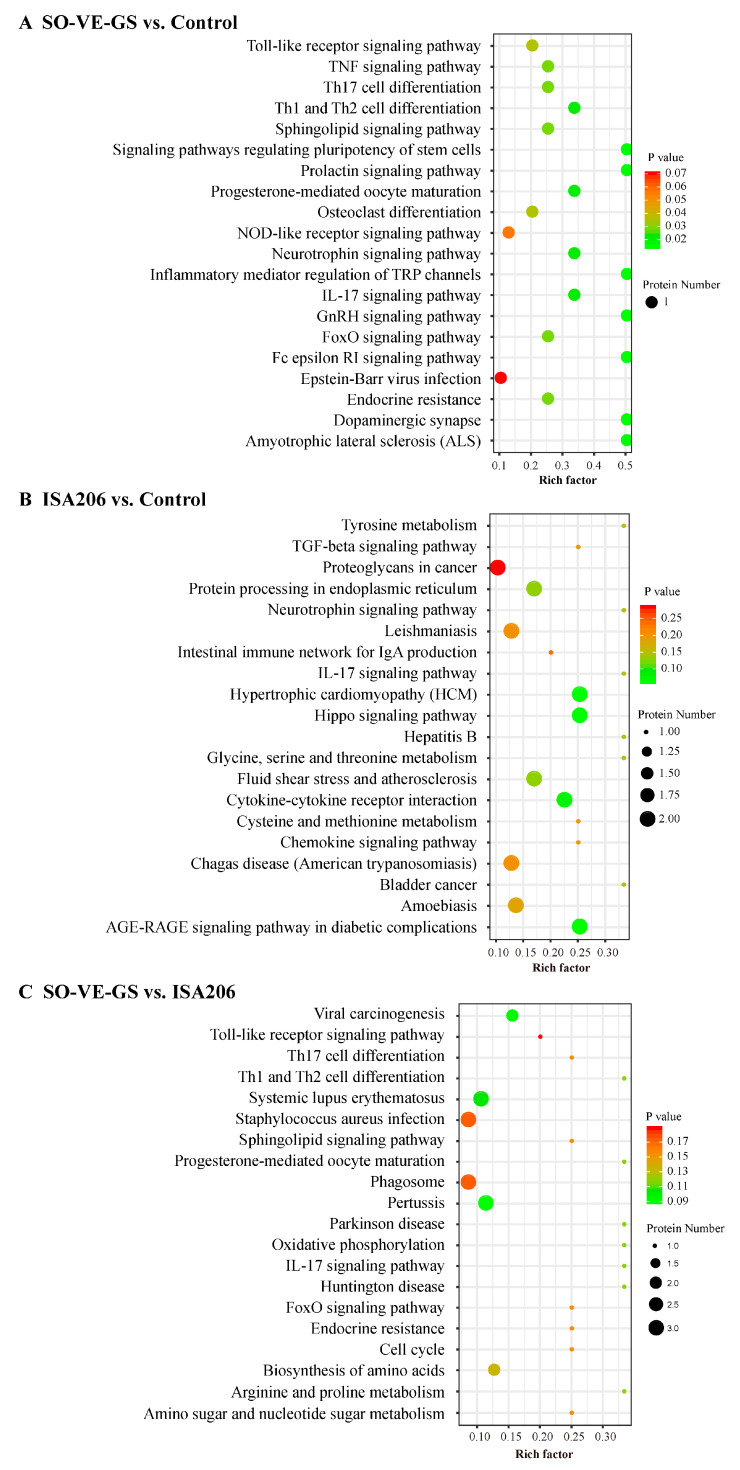
Enrichment of Kyoto Encyclopedia of Genes and Genomes (KEGG) pathways analysis. DEPs of (**A**) SO-VE-GS vs. control, (**B**) ISA 206 vs. control, and (**C**) SO-VE-GS vs. ISA 206 were used for KEGG pathway analysis. The x-axis represents the value of the rich factor. The y-axis represents the enrichment term of the pathways. The size of the dot represents the number of DEPs. The color represents corrected *P* value.

KEGG (Kyoto Encyclopedia of Genes and Genomes) pathway analyses [43] were conducted to comprehensively understand the protein profiles in the different groups. The proteins were classified into six categories by KEGG analysis, which included metabolism (M), genetic information processing (GIP), environmental information processing (EIP), cellular processes (CP), organismal systems (OS), and human disease (HD). The most proteins were identified in the immune system and signal transduction pathways, having 110 and 66 proteins involved, respectively (Figure S5). The top 20 KEGG pathways were used for analyzing the DEPs. Compared to the control group, pathways related to cell differentiation, i.e., differentiation of Th1/Th2 cells, osteoclasts, and Th17 cells, pathways involved in signal transduction, i.e., IL-17 signaling, Toll-like receptor signaling, TNF signaling pathways, and inflammatory mediator regulation of TRP channels were significantly activated by SO-VE-GS (Figure 6A). Interestingly, protein MAPK14 was the common DEP identified in these pathways. In the ISA 206 vs. control comparison, five immune related pathways including intestinal immune network for IgA production, cytokine–cytokine receptor interaction, IL-17 signaling pathway, TGF-beta signaling pathway, and chemokine signaling pathway were enriched; TGF-β1 is a vital protein in these pathways (Figure 6B). Compared with the ISA 206 group, DEPs contained in the SO-VE-GS group were assigned to Toll-like receptor signaling pathway, Th1/Th2 cell differentiation, IL-17 signaling pathway, and Th17 cell differentiation, again, the immune-related pathways focused on protein MAPK14 (Figure 6C).

### 3.8. Interaction Network of DEPs 

**Figure 7:**
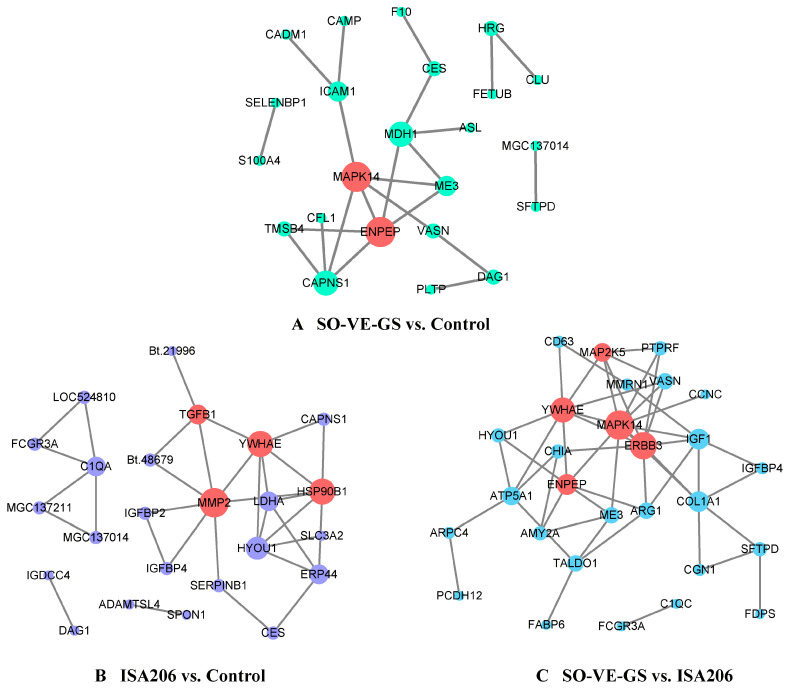
The protein–protein interaction network of DEPs. (**A**) SO-VE-GS vs. control, (**B**) ISA 206 vs. control, and (**C**) SO-VE-GS vs. ISA 206. In the network, nodes represent proteins, and lines represent the predicted associations.

To explore the protein–protein interaction networks, STRING software [44] was adopted. In the SO-VE-GS vs. control comparison, 23 differentially expressed proteins were analyzed and 16 of them constituted an interaction network with 23 nodes and 23 edges. Proteins MAPK14 and ENPEP each interacted with 5 proteins (Figure 7A). In the ISA 206 vs. control comparison, 24 of 47 differentially expressed proteins were detected and 20 of them constituted a network, among these proteins MMP2, YWHAE, HSP90B1, and TGF-β1 interacted with 7, 6, 6, and 4 proteins, respectively (Figure 7B). In the SO-VE-GS vs. ISA 206 comparison, the interaction network was comprised of 28 nodes and 51 edges, and 28 of 49 differentially expressed proteins were detected with 26 proteins connected to nodes (Figure 7C). Proteins MAPK14, ERBB3, YWHAE, ENPEP, and MAP2K5 were found to be important in the interaction network, and interacted with 11, 10, 8, 6, and 5 proteins, respectively.

## 4. Discussion

Hu sheep is an important economic species because of its valuable wool and meat. As the animals are susceptible to FMD, immunization of the sheep with FMD vaccines is compulsory in China in order to control this disease. Most FMD vaccines are typically produced from virus that is generated in suspension cell cultures, then inactivated with binary ethylenimine and formulated with an oil-based adjuvant [45,46]. In China, commercial FMD vaccines are usually adjuvanted with ISA 206, a mineral oil-based adjuvant. The present study showed that the FMD vaccine adjuvanted with ISA 206 induced poor antibody responses. The minimum protective titer against FMDV is considered to be 1:128. Although injection of FMD vaccine adjuvanted with ISA 206 induced protective antibody response in all the animals at 2 and 4 weeks, the antibody titers started to decline at 6 weeks, and 6 of 9 animals had IgG titers less than 1:128 at 8 weeks post immunization (Figure 1). Poor immune responses induced by FMD vaccines were also found in cattle and swine. For example, Dar et al. found that 34% of the cattle challenged with homologous FMDV became persistently infected at 30 days after vaccination with ISA 206 adjuvanted FMD vaccine [45]. Li et al. observed that FMD type O antigen emulsified with adjuvant ISA 206 induced lower antibody titer in pigs [47]. Therefore, searching for potent adjuvants to improve the FMD vaccine is needed. Protective immunity to FMDV is usually attributed to the induction of neutralizing antibodies [48,49]. Thus, for an adjuvant to be effective, it should be able to generate neutralizing antibody responses at higher rates for longer duration. In the present study, a strong correlation was observed between LPB-ELISA IgG titers and neutralizing antibody responses. At 8 weeks post vaccination, the FMD vaccine induced serum IgG titers > 1:128 in 90% of animals (9/10) with SN antibody titers from 1:49 to 1:328 in the SO-VE-GS group while only 33.3% of animals (3/9) had IgG titer more than 1:128 with SN antibody titers less than 1:16 in the ISA 206 group (Figure 1 and Table 1). However, a 6-month duration of antibody titers is needed as administration of the FMD vaccine is recommended every 6 months. However, the declining antibody response was detected in this study 8 weeks after immunization of FMD vaccines with both adjuvants. This paper is the first to report on the dynamic changes of antibody response during the 8 weeks following FMD vaccination in Hu sheep. Therefore, modification of the current vaccine formulation is needed to provide acceptable long-term immunity to FMDV in Hu sheep. The present study was designed to compare only two FMD vaccine formulations, as was done in the study by Selim et al [50]. In order to compare the adjuvant effects of SO-VE-GS and ISA 206 on the FMD vaccine, a group of sheep immunized with FMD antigen without adjuvant should be included.

Different subtypes of IgG, such as IgG1 and IgG2, provide animals with the bulk of immunity against the majority of microbial agents. During T cell-dependent immunity, the major IgG subtypes progressively change [51] and are influenced by T lymphocytes and their produced cytokines. In animals, IL-4 is preferred to switch activated B cells into produce the IgG1 antibody (Th2) and IFN-γ is associated with boosting the IgG2 response (Th1) [52,53,54,55]. The present results showed that SO-VE-GS promoted significantly higher IgG1 and IgG2 than did ISA 206 (Figure 1F,G). The increase of IgG1 and IgG2 is associated with increased IL-4 and IFN-γ (Figure 2), indicating that SO-VE-GS might be activated Th1 and Th2 responses in Hu sheep. Additionally, the pathways identified in Figure 6 seem to suggest that Th17 responses were also activated. The same results were also observed in our previous study where Th1 and Th2 immune responses are activated by SO-VE-GS in mice [32].

To investigate the mechanisms underlying the adjuvant properties of SO-VE-GS, a transcriptomic approach was previously used in a mouse model. This study suggested that the adjuvant properties of SO-VE-GS may be attributed to activation of immune-related genes and pathways [32]. This proteomic technology [56] is based on isotopic labeling and has been applied in several aspects, including production mechanisms [57], growth performance [44], and disease resistance of animals [58,59,60]. However, few studies have looked at its use in vaccine adjuvant research. In this study, TMT (tandem mass tag) was carried out to define relative expression of serum proteins in Hu sheep vaccinated with an FMD vaccine. Protein identification and quantitation showed that 1330 proteins were identified depending on evaluation of peptide matching error, peptide length, protein molecular weight, and protein sequence coverage (Figure 3) [58,61]. When compared with the control group, 2 up- and 7 downregulated DEPs were shared by the two adjuvant groups (Figure 4E,F). The 2 up-regulated proteins were W5Q5J4 (https://www.uniprot.org/uniprot/W5Q5J4) and IL1RAP (https://www.uniprot.org/uniprot/W5QGY1). They were associated with immune response and cytokine GO terms, so we suspect that they might be related to the two adjuvants shown in Figure 2 that promote higher-level cytokines.

GO and KEGG analyses showed that SO-VE-GS and ISA 206 had different enrichment terms (Figure 5 and Figure 6). Compared to the control or ISA 206, SO-VE-GS enriched terms included upregulated cytokine (BP) and Th1/Th2 cell differentiation (OS) in the immune system, which could explain the increase in IgG1, IgG2, IFN-γ, and IL-4 (Figure 1F,G and Figure 2). In addition, the receptor signaling protein activity (MF), Th17 cell differentiation (OS), IL-17 signaling pathway (OS), and Toll-like receptor signaling pathway (OS) were also activated by SO-VE-GS. Among these activated immune related items, DEP MAPK14 (Peptides 1, Unique Peptides 1) was a protein that was commonly shared and significantly upregulated. The interaction network of DEPs further confirmed the significance of MAPK14 in SO-VE-GS (Figure 7A,C). MAPK14 is a mitogen-activated protein kinase (MAPK) (https://www.uniprot.org/uniprot/B7TJ15#function) and plays an important role in regulating the immunological response and the production of specific cytokines and chemokines [62]. Coupled with other related reports, MAPK14 belongs to p38 MAPK, and carried out the majority of p38 MAPK functions [4,63]. It is reported that p38 MAPK is involved in defense against various viral and bacterial infections [64,65,66]. Therefore, MAPK14 might play a key role in SO-VE-GS protection against FMDV in Hu sheep. In ISA 206 adjuvant enriched immune terms (e.g., antigen binding (MF), modulation by virus of host morphology or physiology (BP), cytokine–cytokine receptor interaction (EIP), and TGF-beta signaling pathway (EIP)), TGF-β1 (Peptides 1, Unique Peptides 1) is a vital protein (Figure 7B). TGF-β1 is a transforming growth factor beta-1 proprotein (https://www.uniprot.org/uniprot/P50414). It is reported that TGF-β1 can inhibit production of IFN-γ [67], so significantly higher levels of IFN-γ in the ISA 206 group might be associated with significantly downregulated TGF-β1 (Figure 2A). 

## 5. Conclusions

The immune responses were induced by injection of FMD vaccines adjuvanted with SO-VE-GS and ISA 206 in Hu sheep. SO-VE-GS promoted longer IgG and SN antibodies responses than did ISA 206. In addition, the former stimulated significantly higher production of IgG1, IgG2, IL-4, and IFN-γ than the latter, indicating that Th1 and Th2 immune responses might be activated. Different effects of SO-VE-GS and ISA 206 on the FMD vaccine may be explained by the different proteomic profiles in the two groups. There were 39 and 47 differentially expressed proteins (DEPs) identified in SO-VE-GS compared to the control or ISA 206 group, respectively. In SO-VE-GS vs. control, 3 immune related gene ontology (GO) terms and 8 Kyoto Encyclopedia of Genes and Genomes (KEGG) pathways were detected, while 2 immune related GO terms and 5 KEGG pathways were found in ISA 206 vs. control. GO and KEGG analyses indicated that ‘positive regulation of cytokine secretion’, ‘Th1/Th2 cell differentiation’, and ‘Toll-like receptor signaling pathways’ were obviously enriched in the SO-VE-GS group compared to the other groups. Coupled with protein–protein interaction (PPI) analysis, MAPK14 was suggested a key DEP for SO-VE-GS to activate the immune responses in Hu sheep. Therefore, SO-VE-GS could be a promising adjuvant for an FMD vaccine in Hu sheep. 

## Figures and Tables

**Table 1:** Serum neutralizing (SN) antibody titer.

Group	Animal	SN Antibody Titer	Log_10_ SN Titer
Control	1-1	<1:16	<1.20
	1-2	<1:16	<1.20
	1-3	<1:16	<1.20
	1-4	<1:16	<1.20
	1-5	< 1:16	<1.20
	1-6	< 1:16	<1.20
	1-7	<1:16	<1.20
	1-8	<1:16	<1.20
	1-9	<1:16	<1.20
	1-10	<1:16	<1.20
SO-VE-GS	2-1	1:190	2.27
	2-2	1:256	2.40
	2-3	1:177	2.24
	2-4	1:273	2.43
	2-5	1:97	1.98
	2-6	1:215	2.33
	2-7	1:328	2.51
	2-8	1:49	1.69
	2-9	1:156	2.19
	2-10	1:153	1.72
ISA 206	3-1	<1:16	<1.20
	3-2	<1:16	<1.20
	3-3	<1:16	<1.20
	3-4	<1:16	<1.20
	3-5	<1:16	<1.20
	3-6	<1:16	<1.20
	3-7	<1:16	<1.20
	3-8	<1:16	<1.20
	3-9	<1:16	<1.20

## Supplementary Materials

The following are available online at https://www.mdpi.com/2076-393X/8/2/180/s1, Figure S1: The technical document from Lanzhou Veterinary Research Institute. The relationship between protection level and antibody titer was translated into English, Figure S2: FMDV-IgG titer. Serum FMDV-specific antibody response. (A-D) FMDV-specific IgG titers determined at 2, 4, 6 and 8 weeks post the booster; dotted horizontal line was at IgG titer of 1:128, indicating the minimum protection titer. The values are presented as mean ± SE. Bars with different letters are statistically different (*p* < 0.05), Figure S3: FMDV-SN titer. FMDV-SN titer determined at 8 weeks post the booster. The values are presented as mean ± SE. Bars with different letters are statistically different (*p* < 0.05), Figure S4: Analysis of Hu sheep serum proteome profile by TMT. “Total spectrum” is the total number of the secondary mass spectra, and “Identified spectrum” is the number of the secondary mass spectra after quality control. “Peptide” is the number of the identified peptides, “Unique peptide” is the number of the identified peptides which belong only to a group of proteins, “Protein” is the number of identified proteins, “Protein group number” is the group number of identified proteins, Figure S5: Proteins as classified into five main categories by KEGG analysis: metabolism, genetic information processing, environmental information processing, cellular processes, organismal systems and human diseases. The x-axis indicates the number of proteins within that specific category, Table S1: Protein common names to UniProt accession ID, Table S2: SO-VE-GS vs. Control, ISA_206 vs. Control, and SO-VE-GS vs. ISA 206.
