# Supplementary material for: Enhanced Immune Responses with Serum Proteomic Analysis of Hu Sheep to Foot-and-Mouth Disease Vaccine Emulsified in a Vegetable Oil Adjuvant
Source: Vaccines (Basel). 2020 Apr 15;8(2):180. doi: 10.3390/vaccines8020180 (PMC7349086; doi:10.3390/vaccines8020180)
Supplement: Supplementary file 1 [file vaccines-08-00180-s001.zip › vaccines-733822-xmi-sup1.pdf]

vaccines

MDPI

Article

Enhanced Immune Responses with Serum Proteomic Analysis of Hu Sheep to Foot-and-Mouth Disease Vaccine emulsified in a Vegetable Oil Adjuvant

Xuemei Cui, Yong Wang, Ran Guan, Meiqian Lu, Lijia Yuan, Wei Xu and Songhua Hu *

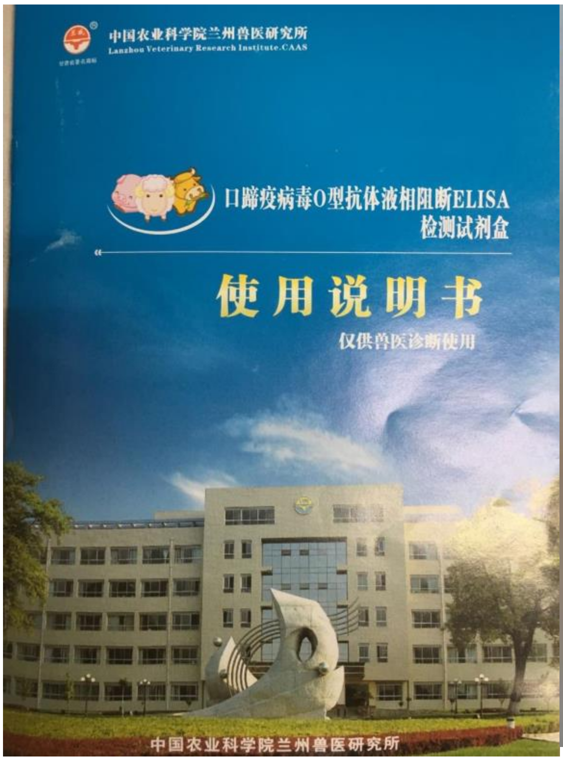

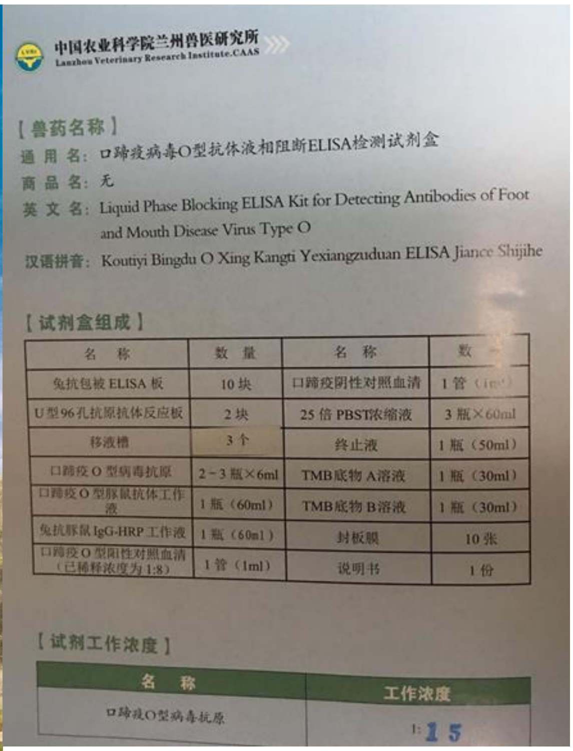

中国农业科学院兰州兽医研究所
Lanzhou Veterinary Research Institute.CAAS

【兽药名称】

通 用 名：口蹄疫病毒O型抗体液相阻断ELISA检测试剂盒

商 品 名：无

英 文 名：Liquid Phase Blocking ELISA Kit for Detecting Antibodies of Foot and Mouth Disease Virus Type O

汉语拼音：Koutiyi Bingdu O Xing Kangti Yexiangzuduan ELISA Jiance Shijihe

【试剂盒组成】

名　称	数　量	名　称	数 [illegible]
兔抗包被 ELISA 板	10 块	口蹄疫阴性对照血清	1 管 [illegible]
U 型 96 孔抗原抗体反应板	2 块	25 倍 PBST浓缩液	3 瓶×60ml
移液槽	3 个	终止液	1 瓶（50ml）
口蹄疫 O 型病毒抗原	2－3 瓶×6ml	TMB底物 A溶液	1 瓶（30ml）
口蹄疫 O 型豚鼠抗体工作液	1 瓶（60ml）	TMB底物 B溶液	1 瓶（30ml）
兔抗豚鼠 IgG-HRP 工作液	1 瓶（60ml）	封板膜	10 张
口蹄疫 O 型阳性对照血清（已稀释浓度为 1:8）	1 管（1ml）	说明书	1 份

【试剂工作浓度】

名　称	工作浓度
口蹄疫O型病毒抗原	1:15

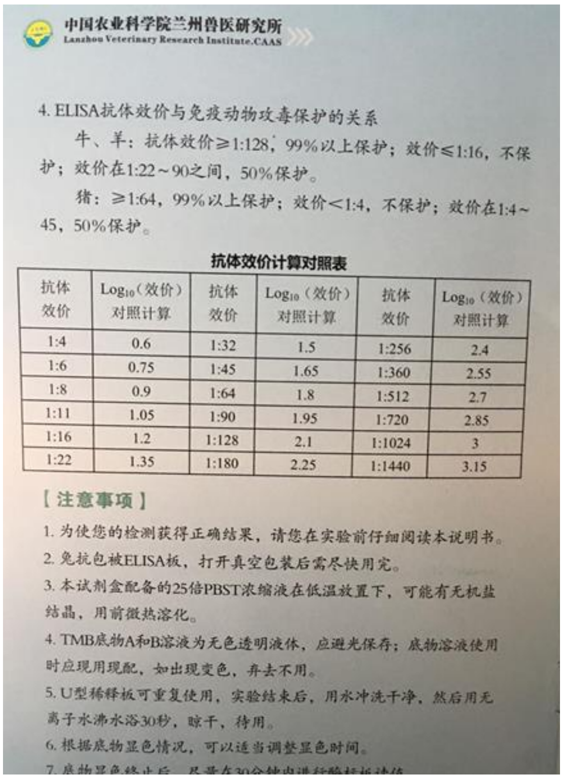

中国农业科学院兰州兽医研究所
Lanzhou Veterinary Research Institute.CAAS

4. ELISA抗体效价与免疫动物攻毒保护的关系

牛、羊：抗体效价≥1:128，99%以上保护；效价≤1:16，不保护；效价在1:22～90之间，50%保护。

猪：≥1:64，99%以上保护；效价<1:4，不保护；效价在1:4～45，50%保护。

抗体效价计算对照表

抗体效价	Log_{10}（效价）对照计算	抗体效价	Log_{10}（效价）对照计算	抗体效价	Log_{10}（效价）对照计算
1:4	0.6	1:32	1.5	1:256	2.4
1:6	0.75	1:45	1.65	1:360	2.55
1:8	0.9	1:64	1.8	1:512	2.7
1:11	1.05	1:90	1.95	1:720	2.85
1:16	1.2	1:128	2.1	1:1024	3
1:22	1.35	1:180	2.25	1:1440	3.15

【注意事项】

1. 为使您的检测获得正确结果，请您在实验前仔细阅读本说明书。
2. 免抗包被ELISA板，打开真空包装后需尽快用完。
3. 本试剂盒配备的25倍PBST浓缩液在低温放置下，可能有无机盐结晶，用前微热溶化。
4. TMB底物A和B溶液为无色透明液体，应避光保存；底物溶液使用时应现用现配，如出现变色，弃去不用。
5. U型稀释板可重复使用，实验结束后，用水冲洗干净，然后用无离子水沸水浴30秒，晾干，待用。
6. 根据底物显色情况，可以适当调整显色时间。
7. 底物显色终止后，[illegible]

Figure S1. The technical document from Lanzhou Veterinary Research Institute. The relationship between protection level and antibody titer was translated into English. The relationship between protection level and antibody titer was described as the manufacturer's instructions (Lanzhou Veterinary Research Institute, Lanzhou, China): sheep and cattle LPB-ELISA antibody titers ≥ 7 log2 (1:128) were considered to indicate > 99% protection, antibody titers ≤ 4 log2 (1:16) no protection, 4.5 ≤ antibody titers ≤ 6.5 log2 (1:22 to 1:90) 50% protection.

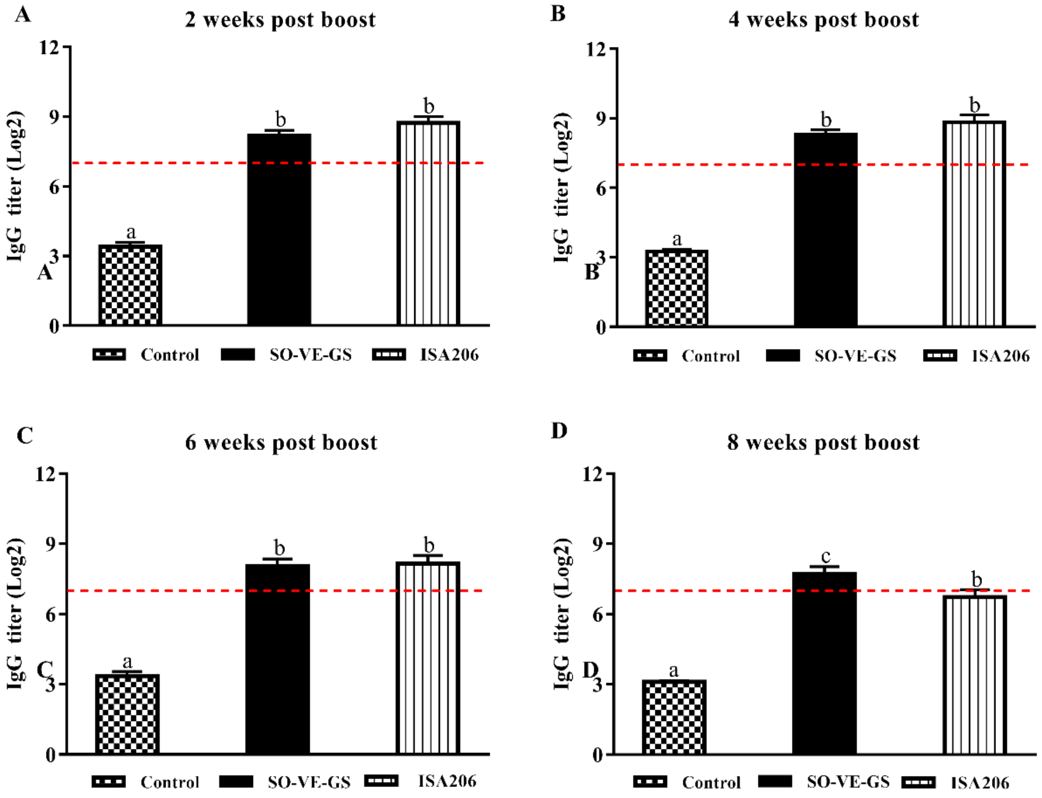

Figure S2. FMDV-IgG titer. Serum FMDV-specific antibody response. (A-D) FMDV-specific IgG titers determined at 2, 4, 6 and 8 weeks post the booster; dotted horizontal line was at IgG titer of 1:128, indicating the minimum protection titer. The values are presented as mean ± SE. Bars with different letters are statistically different ($p < 0.05$).

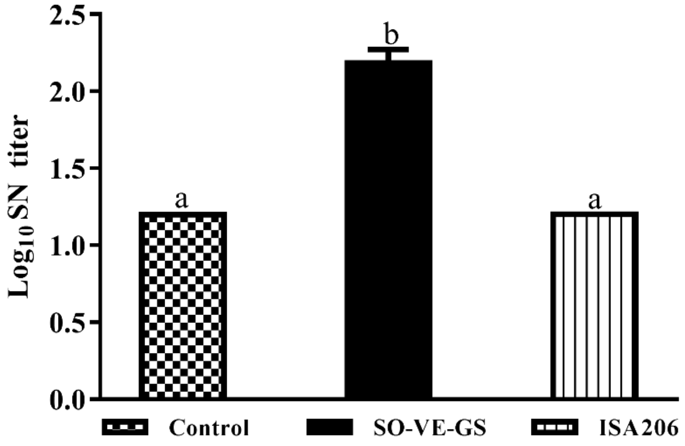

Figure S3. FMDV-SN titer. FMDV-SN titer determined at 8 weeks post the booster. The values are presented as mean ± SE. Bars with different letters are statistically different ($p < 0.05$).

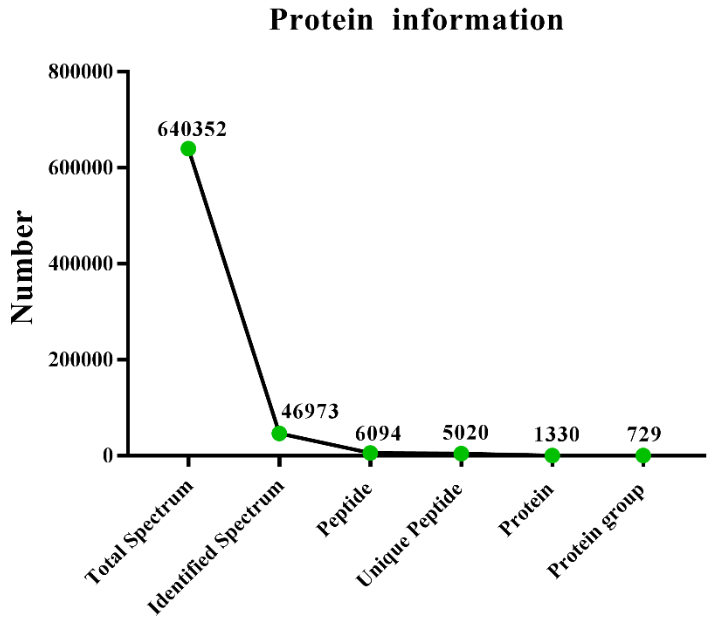

Figure S4. Analysis of Hu sheep serum proteome profile by TMT. "Total spectrum" is the total number of the secondary mass spectra, and "Identified spectrum" is the number of the secondary mass spectra after quality control. "Peptide" is the number of the identified peptides, "Unique peptide" is the number of the identified peptides which belong only to a group of proteins, "Protein" is the number of identified proteins, "Protein group number" is the group number of identified proteins.

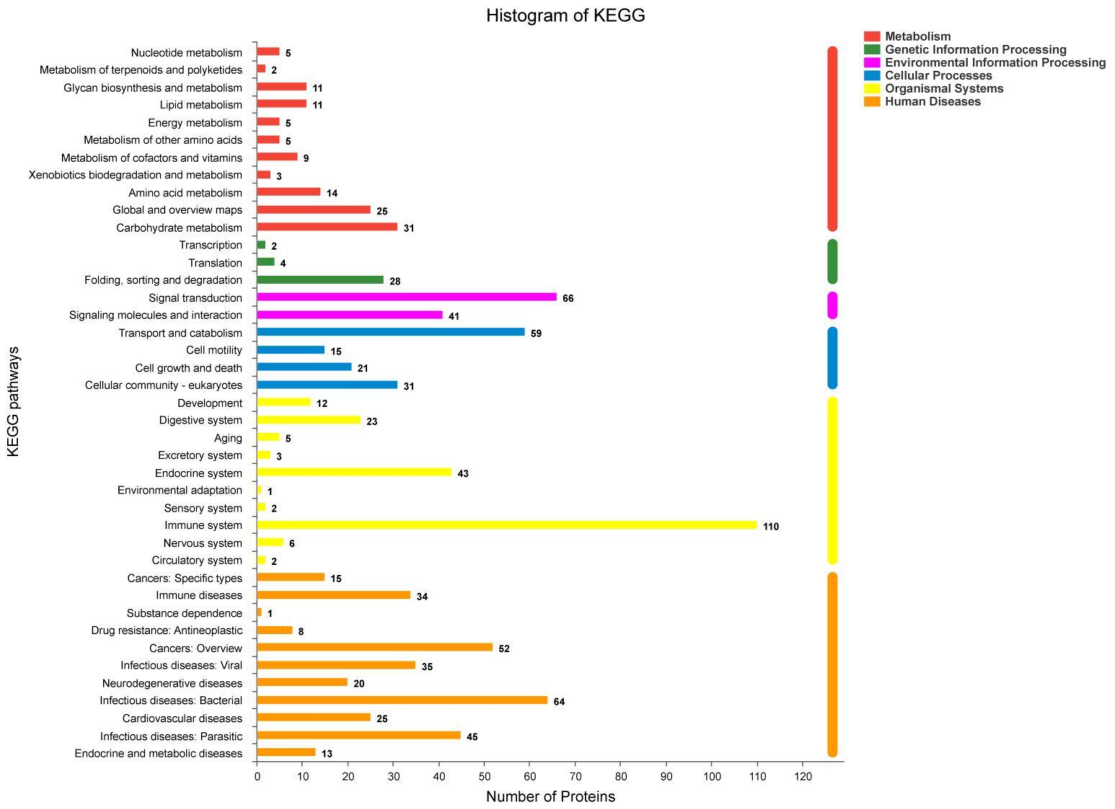

Figure S5. Proteins as classified into five main categories by KEGG analysis: metabolism, genetic information processing, environmental information processing, cellular processes, organismal systems and human diseases. The x-axis indicates the number of proteins within that specific category.

Table S1. Protein common names to UniProt accession ID.

Gene Names (Common Names)	UniProtKB Accession ID
1. IGF1	A0A3Q9U494
2. MAPK14	B7TJ15
3. MMP2	C8BKE4
4. SERPINB1	D8X187
5. CLU	P17698
6. TGFB1	P50414
7. YWHAE	P62262
8. IGFBP4	Q28893
9. IGFBP2	Q29400
10. CFL1	Q6B7M7
11. COL1A1	Q9MZW3
12. PTPRF	W5NQN3
13. S100A4	W5NR63
14. ASL	W5NRA9
15. CAMP	W5NT47
16. CADM1	W5NTW0
17. PCDH12	W5NU41
18. ATP5A1	W5NY50
19. ENPEP	W5P0N0
20. TMSB4	W5P0V0
21. ME3	W5P2A9
22. FDPS	W5P2J9
23. CAPNS1	W5P5G5
24. TALDO1	W5P7X3
25. ARPC4	W5P824

26.	PLTP	W5PB46
27.	SPON1	W5PBA6
28.	F10	W5PD84
29.	C1QC	W5PDQ9
30.	C1QA	W5PDS4
31.	LOC524810	W5PGE9
32.	Bt.21996	W5PGT9
33.	LDHA	W5PIN4
34.	SFTPD	W5PK07
35.	FCGR3A	W5PK31
36.	CGN1	W5PK77
37.	Bt.48679	W5PL47
38.	ERBB3	W5PL95
39.	VASN	W5PLU1
40.	ERP44	W5PMM2
41.	CD63	W5PNU8
42.	CCNC	W5PQC0
43.	HYOU1	W5PSK1
44.	FABP6	W5PUU1
45.	DAG1	W5PVZ9
46.	ARG1	W5PWY0
47.	ICAM1	W5Q263
48.	HSP90B1	W5Q4E1
49.	SLC3A2	W5Q8K4
50.	IGDCC4	W5Q938
51.	CES	W5QA37
52.	MGC137211	W5QA54
53.	MGC137014	W5QA64
54.	MAP2K5	W5QAG1
55.	MMRN1	W5QAK3
56.	AMY2A	W5QBL8
57.	CHIA	W5QE46
58.	MDH1	W5QFQ1
59.	HRG	W5QH50
60.	FETUB	W5QH54
61.	ADAMTSL4	W5QI32
62.	SELENBP1	W5QIK8
63.	IL1RAP	W5QGY1
64.	CADM1	W5NTW0
65.	PAM	W5QAM0
66.	MDH1	W5QFQ1
67.	N/A	C5IJ88
68.	IGFBP-3	C0LQH2
69.	ME3	W5P2A9
70.	N/A	W5NUW3
71.	ERP44	W5PMM2
72.	IGFBP4	Q28893
73.	HSP90B1	W5Q4E1
74.	N/A	W5PGE9
75.	ERBB3	W5PL95
76.	MAP2K5	W5QAG1
77.	FCGR3A	W5PK31
78.	CEMIP	W5Q1S8

© 2020 by the authors. Submitted for possible open access publication under the terms and conditions of the Creative Commons Attribution (CC BY) license (http://creativecommons.org/licenses/by/4.0/).
